# Uric Acid-to-Magnesium Ratio as a Novel Biomarker of Angiographic Coronary Artery Disease Burden: A Retrospective Cross-Sectional Comparative Analysis with the Uric Acid-to-HDL Ratio and Frontal QRS-T Angle in Patients Undergoing Elective Coronary Angiography

**DOI:** 10.3390/jcdd13070342

**Published:** 2026-07-21

**Authors:** Oguz Kaan Kaya, Şükriye Uslu

**Affiliations:** Department of Cardiology, Antalya Training and Research Hospital, University of Health Sciences, 07100 Antalya, Türkiye

**Keywords:** Gensini score, coronary atherosclerosis, angiographic disease severity, magnesium, uric acid, cardiovascular biomarkers, frontal QRS-T angle, risk stratification

## Abstract

**Background:** Non-invasive biomarkers may facilitate early risk stratification in patients with coronary artery disease (CAD). Although the uric acid-to-HDL cholesterol ratio (UHR) has been identified as a novel marker associated with coronary atherosclerosis, the relationship between the uric acid-to-magnesium ratio (UA/Mg ratio) and the angiographic burden of coronary artery disease has not been sufficiently investigated. This study evaluated the association between the UA/Mg ratio and angiographic coronary artery disease burden in patients undergoing elective coronary angiography and compared its discriminative performance with that of the UHR and the frontal QRS-T angle. **Methods:** In this retrospective cross-sectional study, patients who underwent elective coronary angiography between 2022 and 2025 were evaluated. Patients with atrial fibrillation, left ventricular ejection fraction <50%, eGFR < 50 mL/min/1.73 m^2^, severe valvular disease, uric acid-lowering therapy, or acute coronary syndrome were excluded. A total of 351 patients were included in the study and divided into three groups—low (*n* = 117), intermediate (*n* = 117), and high (*n* = 117)—based on their Gensini scores. Independent associations were evaluated using multivariable ordinal logistic regression analysis. Receiver operating characteristic (ROC) analysis was performed to evaluate discrimination of the high Gensini tertile. **Results:** The UA/Mg ratio was strongly correlated with the Gensini score (ρ = 0.699, *p* < 0.001), showing a correlation comparable to that of the UHR (ρ = 0.717, *p* < 0.001) and stronger than that of the frontal QRS-T angle (ρ = 0.546, *p* < 0.001). In the multivariable ordinal logistic regression analysis, the UA/Mg ratio remained independently associated with higher Gensini tertiles (OR = 2.34, 95% CI: 1.23–4.45; *p* = 0.009). In the ROC analysis, the UA/Mg ratio demonstrated excellent discriminative performance for identifying the high Gensini tertile (AUC = 0.907, 95% CI: 0.877–0.938). The AUC of the UA/Mg ratio was comparable to that of the UHR (DeLong *p* = 0.978), whereas it was significantly higher than that of the frontal QRS-T angle (DeLong *p* < 0.001). **Conclusions:** The uric acid-to-magnesium ratio was strongly and independently associated with the burden of angiographic coronary artery disease in patients undergoing elective coronary angiography. The UA/Mg ratio demonstrated discriminative performance comparable to that of the UHR and superior to the frontal QRS-T angle. These findings suggest that the UA/Mg ratio may serve as a complementary biomarker for assessing angiographic coronary artery disease burden. Further prospective studies are warranted to validate its clinical utility.

## 1. Introduction

Despite significant advances in diagnosis and treatment, coronary artery disease (CAD) remains one of the leading causes of mortality and morbidity worldwide [1,2]. Due to the progressive nature of atherosclerosis and the fact that the disease can remain asymptomatic for a long period, accurately assessing the coronary atherosclerotic burden is of critical importance for determining appropriate treatment strategies and preventing long-term cardiovascular events [2,3,4]. Currently, invasive coronary angiography is considered the gold standard method for assessing coronary anatomy, and the Gensini score is one of the most widely used scoring systems for the quantitative determination of coronary atherosclerotic burden [3,5,6,7].

In recent years, there has been growing interest in new biomarkers derived from routine laboratory parameters for the assessment of coronary artery disease. It is considered that composite biomarkers reflecting inflammation, oxidative stress, and metabolic dysregulation in combination may provide stronger prognostic and diagnostic information compared to biochemical parameters used in isolation [8,9,10]. In addition to being the end product of purine metabolism, uric acid has been associated with numerous pathophysiological mechanisms involved in the development of atherosclerosis, such as endothelial dysfunction, oxidative stress, inflammatory cytokine activation, and vascular smooth muscle proliferation [8,9,10,11,12]. However, serum uric acid levels may be of limited value when used alone to assess coronary atherosclerotic burden, as they are influenced by renal function, dietary habits, medication use, and metabolic status [9,10,11,12].

For this reason, in recent years, ratios in which uric acid is evaluated alongside various biochemical parameters are being investigated as more reliable biomarkers [13,14,15]. One of these, the uric acid/HDL cholesterol ratio (UHR), has been identified as a novel cardiovascular risk marker associated with metabolic syndrome, hypertension, diabetes mellitus, and coronary artery disease, as it simultaneously reflects pro-inflammatory and antioxidant mechanisms [13,14,15,16,17,18]. Furthermore, it has been shown that UHR predicts adverse cardiovascular events and is associated with mortality in patients with chronic total coronary occlusion [19,20,21]. However, the fact that HDL cholesterol levels can be influenced by statin therapy, lifestyle changes, and metabolic factors may impose certain limitations on the clinical use of UHR [17,20].

Magnesium, on the other hand, is an essential cation possessing anti-inflammatory, antioxidant, and vasodilator properties that plays a fundamental role in maintaining vascular homeostasis [22,23].

Low serum magnesium levels have been shown to be associated with endothelial dysfunction, arterial stiffness, vascular calcification, insulin resistance, and widespread atherosclerosis [24,25,26,27]. Since uric acid and magnesium exert opposing biological effects on atherosclerosis, the ratio of these two parameters could serve as a novel biomarker that better reflects the balance between vascular inflammation and oxidative stress. However, studies evaluating the relationship between the UA/Mg ratio and coronary atherosclerotic burden are quite limited, and data investigating the clinical value of this ratio—particularly in a homogeneous population undergoing elective coronary angiography—are insufficient [26,27].

On the other hand, electrocardiographic biomarkers are gaining increasing importance in the non-invasive assessment of coronary artery disease. The frontal QRS-T angle is an easily obtainable electrocardiographic parameter reflecting ventricular depolarization and repolarization heterogeneity and has been associated with coronary artery disease, myocardial ischemia, and adverse cardiovascular outcomes [28,29,30,31]. Previous studies have also demonstrated associations between the frontal QRS-T angle and angiographic disease severity, including Gensini and SYNTAX scores [29,30,32,33].

Therefore, the present study aimed to investigate the relationship between the UA/Mg ratio and angiographic coronary atherosclerotic burden in patients undergoing elective coronary angiography, to compare its diagnostic performance with those of the UHR and frontal QRS-T angle, and to determine whether the UA/Mg ratio is independently associated with higher Gensini score beyond traditional cardiovascular risk factors. Furthermore, we aimed to evaluate the potential clinical utility of the UA/Mg ratio as a novel biomarker derived from routine laboratory parameters for assessing coronary atherosclerotic burden because of its ease of application and low cost [3,13,22,28].

## 2. Materials and Methods

### 2.1. Study Design and Setting

This retrospective, single-center study was conducted at the Department of Cardiology, University of Health Sciences Antalya Training and Research Hospital, Antalya, Türkiye. The study was performed in accordance with the principles of the Declaration of Helsinki and was approved by the Clinical Research Ethics Committee of University of Health Sciences Antalya Training and Research Hospital (Approval No. 10/21; Approval Date: 21 May 2026). Due to the retrospective nature of the study, the requirement for written informed consent was waived by the Ethics Committee.

### 2.2. Study Population

Consecutive cases undergoing elective diagnostic angiography between January 2022 and December 2025 were evaluated retrospectively. In line with the aim of the study, only patients undergoing elective coronary angiography were included.

Patients with a history of atrial fibrillation, a left ventricular ejection fraction below 50%, an estimated glomerular filtration rate (eGFR) below 50 mL/min/1.73 m^2^, moderate or severe valvular disease, a diagnosis of acute ST-elevation myocardial infarction (STEMI) or non-ST-elevation myocardial infarction (NSTEMI), or who were receiving uric acid-lowering therapy were excluded from the study. Patients with an eGFR below 50 mL/min/1.73 m^2^ were excluded to minimize the potential confounding effects of impaired renal function on serum uric acid and magnesium levels.

A total of 616 patients were screened. After excluding 265 patients who met the exclusion criteria, 351 patients were included in the final analyses. The enrolled patients were divided into low (*n* = 117), intermediate (*n* = 117), and high (*n* = 117) coronary atherosclerotic burden groups based on the distribution of Gensini scores within the study population, resulting in three equal-sized tertiles.

### 2.3. Clinical and Laboratory Assessment

Demographic characteristics, cardiovascular risk factors, laboratory parameters, and coronary angiography data were obtained from the electronic patient record system. Serum uric acid, HDL cholesterol, magnesium, LDL cholesterol, total cholesterol, triglyceride, creatinine, glucose, and C-reactive protein levels were recorded from routine biochemical analyses.

The uric acid/HDL cholesterol ratio (UHR) was calculated using the following formula:

UHR = (Serum uric acid/HDL cholesterol) × 100

The uric acid/magnesium ratio (UA/Mg) was calculated as follows:

UA/Mg = Serum uric acid (mg/dL)/Serum magnesium (mg/dL)

### 2.4. Electrocardiographic Assessment

In all patients, standard 12-lead electrocardiographic recordings were obtained prior to elective coronary angiography at a paper speed of 25 mm/s and a calibration of 10 mm/mV.

The frontal QRS-T angle was calculated as the absolute angular difference between the frontal QRS axis and the frontal T axis. In cases where the angular difference exceeded 180°, the value was calculated by subtracting it from 360°.

Maximum and minimum P-wave durations were measured manually, and P-wave dispersion was defined as the difference between the maximum and minimum P-wave durations.

QT dispersion was calculated as the difference between the maximum and minimum QT intervals. Corrected QT (QTc) dispersion was defined as the difference between the maximum and minimum QTc intervals, calculated using Bazett’s formula.

### 2.5. Angiographic Assessment and Gensini Score Calculation

Coronary angiograms were independently evaluated by two experienced interventional cardiologists. The severity of coronary artery disease was calculated using the Gensini score.

Coronary stenosis degrees of 25%, 50%, 75%, 90%, 99%, and total occlusion were assigned scores of 1, 2, 4, 8, 16, and 32, respectively. The total Gensini score was calculated by multiplying the obtained scores by weighting factors determined according to the anatomical location of the lesion.

Patients were divided into three groups according to their Gensini scores:

- Low Gensini tertile (*n* = 117).
- Intermediate Gensini tertile (*n* = 117).
- High Gensini tertile (*n* = 117).

### 2.6. Statistical Analysis

Statistical analyses were performed using R software (version 4.5.2; R Foundation for Statistical Computing, Vienna, Austria).

The normality of continuous variables was assessed using the Shapiro–Wilk test. Variables not following a normal distribution were presented as median (interquartile range [IQR]), while categorical variables were presented as counts and percentages.

The Kruskal–Wallis test was used for continuous variables and the chi-square test for categorical variables in comparisons between Gensini groups. Dunn–Bonferroni post hoc analysis was performed for continuous variables where significant differences were detected.

The relationships between the UA/Mg ratio, UHR, frontal QRS-T angle, P-wave dispersion, and QTc dispersion and the Gensini score were evaluated using Spearman correlation analysis.

Multivariable ordinal logistic regression analysis was performed to identify independent predictors of higher Gensini score categories, incorporating age, sex, diabetes mellitus, hypertension, smoking status, low-density lipoprotein (LDL) cholesterol, the uric acid-to-magnesium (UA/Mg) ratio, the uric acid-to-high-density lipoprotein cholesterol (UHR) ratio, and the frontal QRS-T angle. To further evaluate the incremental contribution of the UA/Mg ratio beyond the established UHR, an additional nested model comparison using likelihood ratio testing was performed. Multicollinearity was assessed using the variance inflation factor (VIF).

The diagnostic performance of biomarkers in distinguishing high coronary atherosclerotic burden was evaluated using ROC analysis. The area under the curve (AUC), 95% confidence interval, optimal cut-off value (Youden index), sensitivity, and specificity were calculated. Differences between ROC curves were compared using the DeLong test.

A two-sided *p*-value of <0.05 was considered statistically significant.

### 2.7. Use of Artificial Intelligence Tools

During the preparation of the article, AI-assisted tools were used solely for language editing, grammar checking, and improving the flow of the text. Artificial intelligence tools were not used in data analysis, statistical calculations, the interpretation of results, or scientific decision-making processes. The entire scientific content of the article has been independently reviewed and approved by the authors.

## 3. Results

### 3.1. Study Population and Baseline Characteristics

**Figure 1:**
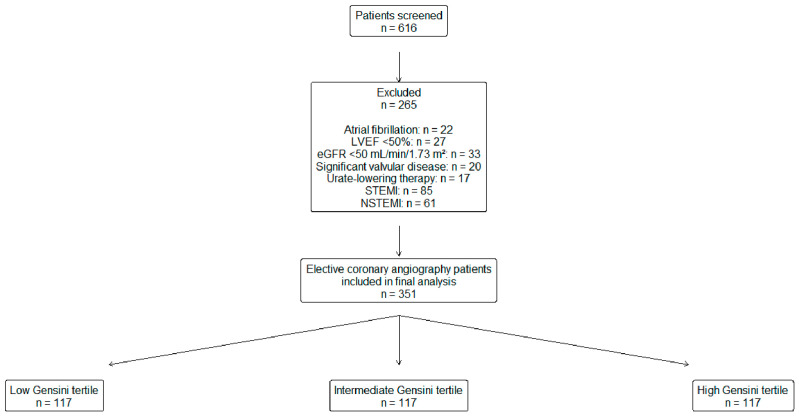
Study flowchart illustrating patient selection and study population. A total of 616 consecutive patients who underwent elective coronary angiography were screened for eligibility. After applying predefined inclusion and exclusion criteria, 351 patients were included in the final analysis and categorized into low (*n* = 117), intermediate (*n* = 117), and high (*n* = 117) Gensini score tertiles. Abbreviations: eGFR, estimated glomerular filtration rate; LVEF, left ventricular ejection fraction; STEMI, ST-segment-elevation myocardial infarction; NSTEMI, non-ST-segment-elevation myocardial infarction.

Between January 2022 and December 2025, a total of 616 patients who underwent elective coronary angiography were screened for eligibility. After applying the predefined exclusion criteria, 265 patients were excluded, and 351 patients were included in the final analysis (Figure 1).

According to the Gensini score, patients were categorized into low (*n* = 117), intermediate (*n* = 117), and high (*n* = 117) coronary atherosclerotic burden groups. Baseline demographic characteristics, cardiovascular risk factors, laboratory findings, including serum creatinine and estimated glomerular filtration rate (eGFR), and electrocardiographic parameters are summarized in Table 1.

As the Gensini score increased, the uric acid-to-magnesium ratio (UA/Mg), uric acid-to-HDL cholesterol ratio (UHR), and frontal QRS-T angle progressively increased (all *p* < 0.001). QTc dispersion also differed significantly across Gensini tertiles (*p* = 0.002). In contrast, although P-wave dispersion showed an overall difference among the tertiles in the Kruskal–Wallis analysis (*p* = 0.001), it was not significantly correlated with the continuous Gensini score in the Spearman analysis (ρ = 0.065, *p* = 0.227).

### 3.2. Correlation Analysis

Spearman correlation analysis demonstrated a strong positive correlation between the UA/Mg ratio and Gensini score (ρ = 0.699, *p* < 0.001). Similarly, UHR showed a strong positive correlation with Gensini score (ρ = 0.717, *p* < 0.001), whereas the frontal QRS-T angle demonstrated a moderate positive correlation (ρ = 0.546, *p* < 0.001).

In contrast, no significant association was observed between P-wave dispersion and Gensini score (ρ = 0.065, *p* = 0.227). QTc dispersion showed a weak but statistically significant positive correlation with Gensini score (ρ = 0.143, *p* = 0.007) (Table 2).

**Figure 2:**
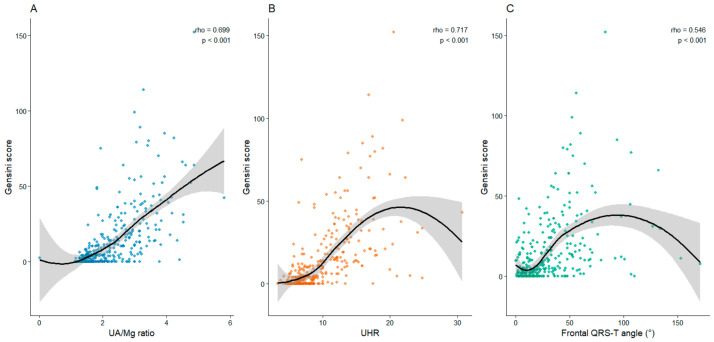
Correlation between biochemical/electrocardiographic markers and angiographic coronary atherosclerotic burden. Scatter plots illustrate the relationships between (**A**) UA/Mg ratio, (**B**) UHR, and (**C**) frontal QRS-T angle and Gensini score. Solid curves represent locally weighted regression (LOESS) trends, and shaded areas indicate the corresponding 95% confidence intervals. Abbreviations: LOESS, locally estimated scatterplot smoothing.

The relationships between UA/Mg ratio, UHR, frontal QRS-T angle, and Gensini score are illustrated in Figure 2.

### 3.3. Multivariable Ordinal Logistic Regression Analysis

Multivariable ordinal logistic regression analysis demonstrated that the UA/Mg ratio (OR = 2.34, 95% CI: 1.23–4.45; *p* = 0.009), UHR (OR = 1.27, 95% CI: 1.10–1.47; *p* < 0.001), frontal QRS-T angle (OR = 1.015, 95% CI: 1.004–1.026; *p* = 0.007), male sex (OR = 0.43, 95% CI: 0.25–0.71; *p* = 0.001), and diabetes mellitus (OR = 1.95, 95% CI: 1.19–3.20; *p* = 0.008) were independently associated with higher Gensini score categories (Table 3). Sensitivity analyses using a partial proportional odds model yielded findings that were broadly consistent with those of the primary ordinal logistic regression analysis.

In an additional nested model comparison, inclusion of the UA/Mg ratio significantly improved overall model fit beyond a model containing the established UHR (likelihood ratio χ^2^ = 7.20, *p* = 0.007), with a corresponding reduction in the Akaike information criterion (562.4 vs. 557.2), supporting an incremental contribution of the UA/Mg ratio to overall model performance.

The proportional odds assumption was formally evaluated using the Brant test. Although the global test suggested some deviation from the proportional odds assumption, the additional partial proportional odds model yielded findings broadly consistent with those of the primary analysis.

No evidence of multicollinearity was observed, with variance inflation factor (VIF) values below 5 for all variables.

### 3.4. Diagnostic Performance of Biomarkers

ROC analysis demonstrated excellent discriminatory performance of the UA/Mg ratio for identifying high coronary atherosclerotic burden (AUC = 0.907, 95% CI: 0.877–0.938). UHR showed a comparable diagnostic performance with an AUC of 0.908 (95% CI: 0.873–0.942).

In contrast, the frontal QRS-T angle demonstrated a lower diagnostic performance, with an AUC of 0.769 (95% CI: 0.713–0.825) (Table 4).

According to the Youden index, the optimal cut-off values were 2.22 for UA/Mg ratio (sensitivity 87.2%, specificity 81.5%), 10.12 for UHR (sensitivity 82.9%, specificity 91.0%), and 22.5° for frontal QRS-T angle (sensitivity 78.6%, specificity 71.8%).

**Figure 3:**
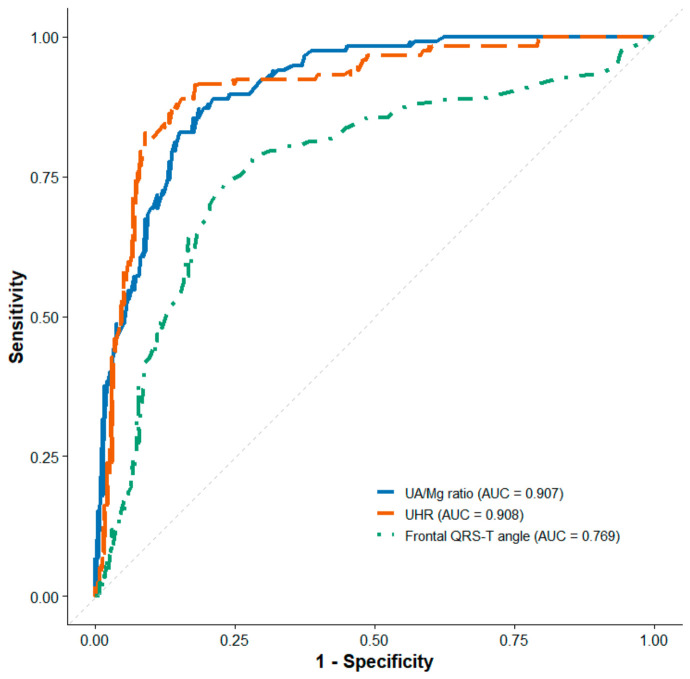
Receiver operating characteristic (ROC) curves of the uric acid-to-magnesium ratio (UA/Mg), uric acid-to-high-density lipoprotein cholesterol ratio (UHR), and frontal QRS-T angle for identifying high coronary atherosclerotic burden. The ROC curves demonstrate the diagnostic performance of UA/Mg ratio, UHR, and frontal QRS-T angle for discriminating patients with high angiographic coronary atherosclerotic burden. Both biochemical markers showed superior discriminatory ability compared with the frontal QRS-T angle, whereas UA/Mg ratio and UHR exhibited comparable overall performance. Abbreviations: ROC, receiver operating characteristic; UA/Mg, uric acid-to-magnesium ratio; UHR, uric acid-to-high-density lipoprotein cholesterol ratio.

The ROC curves of the three investigated biomarkers are presented in Figure 3.

Pairwise comparison of ROC curves using the DeLong test showed no significant difference between UA/Mg ratio and UHR (*p* = 0.978), whereas UA/Mg ratio performed significantly better than the frontal QRS-T angle (*p* < 0.001).

### 3.5. Pairwise Comparisons Across Gensini Tertiles

Dunn–Bonferroni post hoc analysis demonstrated significant differences in the UA/Mg ratio between the low and intermediate Gensini groups (adjusted *p* = 0.011), the low and high groups (adjusted *p* < 0.001), and the intermediate and high groups (adjusted *p* < 0.001).

Similarly, UHR and frontal QRS-T angle differed significantly across all pairwise comparisons between the three Gensini tertiles (all adjusted *p* < 0.001).

**Figure 4:**
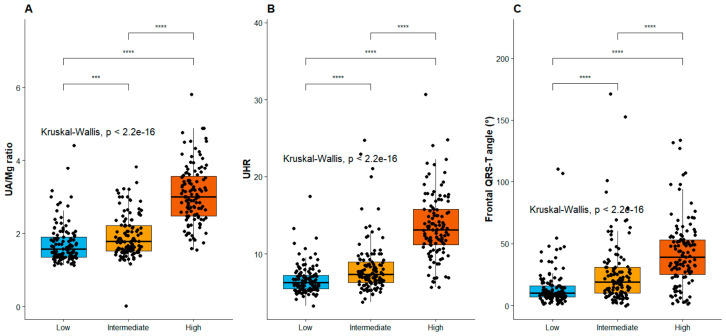
Distribution of UA/Mg ratio, UHR, and frontal QRS-T angle according to Gensini score tertiles. Box-and-whisker plots demonstrate progressive increases in (**A**) UA/Mg ratio, (**B**) UHR, and (**C**) frontal QRS-T angle across low, intermediate, and high Gensini score tertiles. Boxes represent the interquartile range, horizontal lines indicate the median, whiskers represent 1.5× the interquartile range, and individual points represent outliers. Overall comparisons were performed using the Kruskal–Wallis test followed by Dunn–Bonferroni post hoc analysis. Horizontal brackets indicate statistically significant pairwise comparisons. Blue, yellow, and orange boxplots represent the low, intermediate, and high Gensini score tertile groups, respectively. *** *p* < 0.001; **** *p* < 0.0001.

The distributions of UA/Mg ratio, UHR, and frontal QRS-T angle across Gensini score tertiles are shown in Figure 4.

## 4. Discussion

### 4.1. Principal Findings

In this retrospective, single-center study, we demonstrated that the uric acid/magnesium (UA/Mg) ratio is strongly and independently associated with the angiographic burden of coronary artery disease in patients undergoing elective coronary angiography. The main findings of the study can be summarized as follows: first, the UA/Mg ratio showed a strong positive correlation with the Gensini score. Secondly, the UA/Mg ratio increased in a stepwise manner across the low, medium, and high Gensini score groups and showed significant differences in all pairwise group comparisons. Thirdly, in the multivariable ordinal logistic regression analysis, the UA/Mg ratio remained independently associated with higher Gensini categories, alongside traditional cardiovascular risk factors, UHR, and the frontal QRS-T angle. Fourthly, in the ROC analysis, the UA/Mg ratio demonstrated excellent discriminative performance for identifying a high coronary atherosclerotic burden, and this performance was found to be comparable to that of the established UHR. While supporting previous Gensini and UHR studies, these findings suggest that the UA/Mg ratio may represent a complementary biomarker for assessing angiographic coronary artery disease burden [3,6,13,19].

### 4.2. Interpretation of the UA/Mg Ratio in Coronary Atherosclerotic Burden

Although uric acid was long considered merely the end product of purine metabolism, current evidence indicates that it may play an active role in the atherosclerotic process. Hyperuricemia may facilitate the development of atherosclerosis through mechanisms such as reduced endothelial nitric oxide bioavailability, increased oxidative stress, vascular smooth muscle proliferation, inflammatory cytokine activation, and platelet activation [8,9,10,11,12]. However, when serum uric acid levels are evaluated in isolation, they can be influenced by numerous variables, such as renal function, diet, metabolic status, and medication use. Therefore, composite ratios in which uric acid is evaluated alongside various biochemical parameters may be more meaningful than individual parameters in reflecting coronary atherosclerotic burden [13,14,15]. Recent evidence also highlights the complex interplay among inflammation, oxidative stress, metabolic dysregulation, and vascular injury in the development of coronary atherosclerosis, further supporting the rationale for composite biomarkers integrating multiple biological pathways [34,35,36].

Magnesium, on the other hand, is an essential electrolyte that plays a protective role in vascular biology. Experimental and clinical data indicate that magnesium deficiency is associated with endothelial dysfunction, arterial stiffness, inflammation, oxidative stress, and vascular calcification [22,23,24,25]. Furthermore, low magnesium intake has been shown to be associated with the development of atherosclerotic lesions and cardiovascular mortality [26,27]. In this context, the UA/Mg ratio can be considered a composite index that reflects both the proinflammatory and pro-oxidant effects of uric acid and the reduction in the vascular protective effects of magnesium.

In the present study, the finding that the UA/Mg ratio showed a strong correlation with the Gensini score and remained independently associated with higher Gensini categories supports this biological basis. In particular, the fact that the UA/Mg ratio shows a progressive increase across the low, medium, and high Gensini groups suggests that this ratio may reflect not only the presence of the disease but also the severity of the angiographic disease burden.

### 4.3. Comparison with the Uric Acid-to-HDL Ratio

In recent years, UHR has been increasingly investigated as a novel biomarker associated with cardiometabolic risk, metabolic syndrome, diabetes mellitus, hypertension, and coronary artery disease [13,14,15,16,17,18]. Recent studies have reported that UHR is associated with the presence of coronary artery disease, the Gensini score, chronic total occlusion, and long-term cardiovascular events [19,20,21].

In our study, UHR was one of the parameters showing the strongest correlation with the Gensini score and was independently associated with higher Gensini score categories. This finding is consistent with the existing literature. However, the principal contribution of the present study is not merely that the UA/Mg ratio demonstrated discriminative performance comparable to that of the established UHR. In an additional nested model comparison, inclusion of the UA/Mg ratio significantly improved overall model fit beyond a model containing the established UHR (likelihood ratio χ^2^ = 7.20, *p* = 0.007; AIC: 562.4 vs. 557.2), indicating an incremental contribution of the UA/Mg ratio to overall model performance. Although the ROC analysis showed comparable discriminative performance for the UA/Mg ratio (AUC = 0.907) and the UHR (AUC = 0.908), these findings suggest that the UA/Mg ratio may provide complementary information beyond the established UHR while reflecting different biological mechanisms related to coronary atherosclerosis.

Since HDL cholesterol levels can be influenced by various factors—including statin therapy, lifestyle changes, and metabolic syndrome—the UA/Mg ratio may offer complementary information in selected patient populations.

### 4.4. Electrocardiographic Markers and Frontal QRS-T Angle

The frontal QRS-T angle is an easily calculable electrocardiographic parameter that reflects the discordance between ventricular depolarization and repolarization vectors [28,29,30]. An increased frontal QRS-T angle has been shown to be associated with myocardial ischemia, ventricular repolarization heterogeneity, arrhythmic risk, and adverse cardiovascular outcomes [30,31,32]. Furthermore, it has been reported that the frontal QRS-T angle is associated with Gensini and SYNTAX scores in stable coronary artery disease [29,30,33].

In the present study, the frontal QRS-T angle showed a moderately strong correlation with the Gensini score and remained independently associated with higher Gensini categories in the multivariable ordinal logistic regression analysis. However, in the ROC analysis, the discriminatory performance of the frontal QRS-T angle was found to be lower than that of UA/Mg and UHR. Therefore, the frontal QRS-T angle should be considered a non-invasive parameter that complements biochemical markers, rather than a strong biomarker on its own.

As for P-wave dispersion and QTc dispersion, the results are more limited. QTc dispersion showed a weak but significant correlation with the Gensini score, whereas P-wave dispersion did not show a significant correlation with the continuous Gensini score despite showing an overall difference across tertiles. These findings suggest that electrocardiographic repolarization parameters may be associated with coronary atherosclerotic burden but provide more limited information compared to biochemical markers such as UA/Mg and UHR.

### 4.5. Clinical Implications

The findings of this study are of importance from several clinical perspectives. First, the UA/Mg ratio can be readily calculated from routinely available laboratory parameters without requiring additional testing. Patients with a high UA/Mg ratio prior to elective coronary angiography may have a greater angiographic coronary atherosclerotic burden [2,3]. Secondly, the fact that the UA/Mg ratio demonstrates diagnostic performance comparable to UHR indicates that this new index could make a significant contribution to the existing biomarker literature [13,19]. Thirdly, the superior performance of the UA/Mg ratio regarding the frontal QRS-T angle suggests that biochemical markers may provide stronger risk classification than electrocardiographic parameters [22,29].

However, the UA/Mg ratio should be considered not as a test to replace coronary angiography, but as a complementary biomarker to be used alongside traditional risk factors, clinical assessment, and other non-invasive tests. Furthermore, the proposed cut-off value of 2.22 should be considered hypothesis-generating and requires prospective validation before routine clinical implementation.

### 4.6. Strengths of the Study

This study has several strengths. First, the study was conducted exclusively on patients undergoing elective coronary angiography; patients with STEMI and NSTEMI were excluded to create a more homogeneous population. Secondly, significant confounding factors such as atrial fibrillation, LVEF < 50%, eGFR < 50 mL/min/1.73 m^2^, severe valvular disease, and the use of uric acid-lowering therapy were excluded. Thirdly, the Gensini score was classified into low, medium, and high tertiles with equal numbers of patients. Finally, the study employed a comprehensive statistical approach including multivariable ordinal logistic regression, sensitivity analyses using a partial proportional odds model, ROC analysis, VIF assessment, and Dunn–Bonferroni post hoc analyses.

## 5. Limitations

This study has certain limitations. First, the study has a retrospective and single-center design; therefore, a causal relationship cannot be established. Secondly, body weight and height were not systematically available in the retrospective electronic medical records. Consequently, body mass index (BMI) could not be reliably calculated, and subgroup analyses according to overweight or obesity status could not be performed. Thirdly, detailed information regarding chronic medication use—including diuretics, SGLT2 inhibitors, statins, antihypertensive drugs, magnesium supplementation, and other metabolic therapies—was not consistently available because of the retrospective study design. Since these medications may influence serum HDL cholesterol, uric acid, and magnesium levels, residual medication-related confounding cannot be excluded. In addition, several established inflammatory and metabolic biomarkers, including the neutrophil-to-lymphocyte ratio, monocyte-to-HDL ratio, triglyceride–glucose index, and fibrinogen, were not routinely available, precluding direct comparisons with the UA/Mg ratio. Fourthly, serum magnesium and uric acid levels were based on single-point measurements; serial measurements were not available. Furthermore, the ROC analyses were performed using the same study population from which the associations were derived; therefore, the reported discriminative performance may be overestimated and requires validation in independent cohorts. Fifthly, advanced imaging modalities such as intravascular ultrasound, optical coherence tomography, or functional ischemia assessment were not used. Finally, because the study population consisted exclusively of patients undergoing elective coronary angiography, the findings cannot be generalized to patients with acute coronary syndrome.

## 6. Conclusions

In conclusion, the UA/Mg ratio was found to be strongly and independently associated with the burden of angiographic coronary artery disease in patients undergoing elective coronary angiography. The UA/Mg ratio demonstrated excellent discriminative performance for identifying a high angiographic coronary artery disease burden, exhibited diagnostic accuracy comparable to the established UHR, and provided incremental information beyond the established UHR by significantly improving model fit. It also showed significantly superior discriminative performance compared with the frontal QRS-T angle. These findings suggest that the UA/Mg ratio may serve as a complementary biomarker for assessing coronary atherosclerotic burden that is easy to apply, low-cost, and integrable into clinical practice. Prospective, multicenter studies with larger sample sizes are required to confirm these findings.

## Figures and Tables

**Table 1:** Baseline clinical, laboratory, electrocardiographic, and angiographic characteristics according to Gensini score tertiles.

Characteristic	Low (*n* = 117)	Intermediate (*n* = 117)	High (*n* = 117)	*p* Value
Clinical characteristics				
Age, years	57.00 (50.00–65.00)	62.00 (54.00–68.00)	61.00 (54.00–67.00)	0.008
Male sex, *n* (%)	51 (44%)	74 (63%)	92 (79%)	<0.001
Diabetes mellitus, *n* (%)	33 (28%)	46 (39%)	61 (52%)	<0.001
Hypertension, *n* (%)	55 (47%)	72 (62%)	74 (63%)	0.022
Current smoking, *n* (%)	44 (38%)	53 (45%)	64 (55%)	0.032
Laboratory parameters				
LDL cholesterol, mg/dL	125.00 (105.00–153.00)	112.00 (98.00–151.00)	113.00 (90.00–149.00)	0.044
HDL cholesterol, mg/dL	56.00 (52.00–61.00)	51.00 (45.00–58.00)	42.00 (36.00–50.00)	<0.001
Uric acid, mg/dL	3.50 (3.10–4.10)	3.70 (3.30–4.50)	5.40 (4.60–6.70)	<0.001
Magnesium, mg/dL	2.20 (2.10–2.30)	2.10 (2.00–2.20)	1.80 (1.70–1.90)	<0.001
Creatinine, mg/dL	0.85 (0.78–0.99)	0.92 (0.83–1.06)	0.97 (0.85–1.10)	<0.001
eGFR, mL/min/1.73 m^2^	88.00 (76.00–95.00)	82.00 (73.00–89.00)	83.00 (72.00–90.00)	0.031
Uric acid-to-HDL ratio	6.29 (5.54–7.22)	7.38 (6.32–9.00)	13.14 (11.22–15.76)	<0.001
Uric acid-to-magnesium ratio	1.57 (1.35–1.90)	1.78 (1.52–2.21)	3.00 (2.47–3.56)	<0.001
Electrocardiographic parameters				
Frontal QRS-T angle, degrees	10.00 (7.00–16.00)	19.00 (10.00–31.00)	39.00 (25.00–53.00)	<0.001
P-wave dispersion, ms	40.00 (35.00–40.00)	40.00 (40.00–40.00)	40.00 (40.00–40.00)	0.001
QTc dispersion, ms	67.00 (56.00–77.00)	75.00 (64.00–85.00)	72.00 (61.00–85.00)	0.002
Angiographic parameter				
Gensini score	0.00 (0.00–0.00)	4.50 (3.00–8.00)	27.00 (17.50–42.00)	<0.001

Data are presented as median (Q1–Q3) or *n* (%). Continuous variables were compared using the Kruskal–Wallis test and categorical variables using Pearson’s chi-squared test. Abbreviations: eGFR, estimated glomerular filtration rate; HDL, high-density lipoprotein; LDL, low-density lipoprotein; QTc, corrected QT interval.

**Table 2:** Spearman correlation analysis between candidate markers and Gensini score.

Marker	Spearman’s Rho	*p*-Value
Uric acid-to-magnesium ratio	0.699	<0.001
Uric acid-to-HDL ratio	0.717	<0.001
Frontal QRS-T angle	0.546	<0.001
QTc dispersion	0.143	0.007
P-wave dispersion	0.065	0.227

Data are presented as Spearman correlation coefficients. Spearman rank correlation analysis was used to evaluate associations between candidate markers and the Gensini score. HDL, high-density lipoprotein; QTc, corrected QT interval; QRS-T, QRS-T angle.

**Table 3:** Multivariable ordinal logistic regression analysis for higher Gensini tertiles.

Variable	Odds Ratio (95% CI)	*p* Value
Uric acid-to-magnesium ratio (UA/Mg)	2.34 (1.23–4.45)	0.009
Uric acid-to-HDL ratio (UHR)	1.27 (1.10–1.47)	<0.001
Frontal QRS-T angle	1.015 (1.004–1.026)	0.007
Age	1.02 (0.99–1.04)	0.167
Male sex	0.43 (0.25–0.71)	0.001
Diabetes mellitus	1.95 (1.19–3.20)	0.008
Hypertension	1.21 (0.75–1.95)	0.443
Current smoking	1.18 (0.72–1.93)	0.522
LDL cholesterol	1.001 (0.995–1.008)	0.644

Data are presented as odds ratios (OR) with 95% confidence intervals derived from multivariable ordinal logistic regression. Age, hypertension, smoking status, and LDL cholesterol levels were not independently associated with Gensini score.

**Table 4:** Diagnostic performance of candidate biomarkers for identifying patients with high angiographic coronary artery disease burden.

Biomarker	AUC (95% CI)	Optimal Cut-Off	Sensitivity (%)	Specificity (%)
Uric acid-to-HDL ratio (UHR)	0.908 (0.873–0.942)	10.12	82.9	91.0
Uric acid-to-magnesium ratio (UA/Mg)	0.907 (0.877–0.938)	2.22	87.2	81.5
Frontal QRS-T angle	0.769 (0.713–0.825)	22.5°	78.6	71.8

AUC, area under the receiver operating characteristic curve; CI, confidence interval. Optimal cut-off values were determined using the Youden index.

## Data Availability

The data supporting the findings of this study are available within the article. Additional anonymized data are available from the corresponding author upon reasonable request, subject to institutional and ethical regulations.
